# Bacteria‐derived ferrichrome inhibits tumor progression in sporadic colorectal neoplasms and colitis‐associated cancer

**DOI:** 10.1186/s12935-020-01723-9

**Published:** 2021-01-06

**Authors:** Takuya Iwama, Mikihiro Fujiya, Hiroaki Konishi, Hiroki Tanaka, Yuki Murakami, Takehito Kunogi, Takahiro Sasaki, Keitaro Takahashi, Katsuyoshi Ando, Nobuhiro Ueno, Shin Kashima, Kentaro Moriichi, Hiroki Tanabe, Toshikatsu Okumura

**Affiliations:** 1 Division of Gastroenterology and Hematology/Oncology, Department of Medicine, Asahikawa Medical University, 2-1-1-1, Midorigaoka, Hokkaido 078-8510 Asahikawa, Japan; 2 Department of Legal Medicine, Asahikawa Medical University, Asahikawa, Japan

**Keywords:** Ferrichrome, Organoid, Colorectal cancer, Probiotics, DDIT3

## Abstract

**Background:**

Colorectal cancers develop through several pathways, including the adenoma–carcinoma sequence and colitis-associated carcinogenesis. An altered intestinal microflora has been reported to be associated with the development and progression of colorectal cancer via these pathways. We identified *Lactobacillus casei*-derived ferrichrome as a mediator of the bacterial anti-tumor effect of colorectal cancer cells through the upregulation of DDIT3. In this study, we investigated the anti-tumor effects of ferrichrome on precancerous conditions and cancer cells associated with sporadic as well as colitis-associated colorectal cancer.

**Methods:**

SRB and MTT assays were performed to assess growth inhibition in vitro. Eighteen organoids were prepared from biopsy specimens obtained by colonoscopy. An AOM-DSS carcinogenesis model and xenograft model of colorectal cancer cells were generated for the assessment of the tumor suppressive effect of ferrichrome in vivo.

**Results:**

Ferrichrome inhibited the cell growth of colorectal cancer cells in vitro and in in vivo xenograft models. Ferrichrome exerted a strong tumor-suppressive effect that was superior to that of currently available anti-tumor agents, including 5-FU and cisplatin, both in vitro and in vivo. The tumor-suppressive effect of the combination of ferrichrome and 5-FU was superior to that of single treatment with either drug. The tumor suppressive effects of ferrichrome were confirmed through the upregulation of DDIT3 in patient-derived organoids of adenoma and carcinoma. Ferrichrome inhibited the tumor progression in the AOM-DSS model while exhibiting no anti-inflammatory effect in the DSS-colitis model, suggesting that ferrichrome inhibited cancer cells, but not a precancerous condition, via the colitis-associated pathway.

**Conclusions:**

Ferrichrome exerts a tumor suppressive effect on precancerous conditions and cancer cells associated with sporadic as well as colitis-associated colorectal cancer. The anti-tumor effect of ferrichrome was mediated by the upregulation of DDIT3, and was superior to that of 5-FU or cisplatin. These results suggest that *Lactobacillus brevis*-derived ferrichrome may be a candidate anti-tumor drug for the treatment of colorectal neoplasms.

## Background

The outcomes of colorectal cancer have been improving with advances in chemotherapy, including the developments of molecular-targeted therapy and new drug delivery systems [1–3]. However, because baseline anti-tumor drugs, such as 5-fluorouracil (5-FU) and cisplatin, are not suitable for elderly patients and cases with comorbidities [4–6], the five-year survival rate of advanced colorectal cancer has remained low [7] due to the low completion rate of chemotherapy [8]. Novel anti-tumor drugs with a strong anti-tumor effect as well as safety are therefore urgently needed.

Several representative pathways, including the adenoma-carcinoma sequence and colitis-associated pathways, are involved in the development and progression of colorectal cancer [9, 10]. Alteration of the *adenomatous polyposis coli* (*APC*) gene is an initial event in adenoma-carcinoma sequence, followed by a *k-ras* gene mutation and subsequently alterations of the *p53* and *Deleted in Colorectal Cancer* (*DCC*) genes [11]. In contrast, the alteration of the p53 function is thought to be an initial event in the colitis-associated pathway, followed by a *k-ras* gene mutation and alteration of the *APC* gene [12], suggesting that the process of cancer development differs among these pathways.

An altered intestinal microflora is associated with the development and progression of colorectal cancer in both the adenoma-carcinoma sequence and colitis-associated pathways [13, 14], suggesting the importance of maintaining homeostasis of the intestinal microflora. Probiotics are live microorganisms that confer a health benefit on the consumer when they are administered in adequate amounts [15] and help maintain intestinal homeostasis [16]. The anti-tumor effect obtained from probiotic bacteria, including Biffidobacterium and Lactobacillus species, in vitro and in vivo, have been shown, suggesting that probiotics are potentially useful for cancer treatment with fewer adverse events than conventional therapies, although the mechanisms have yet to be explored.

We recently reported ferrichrome as an anti-tumor molecule derived from *L. casei American Type Culture. Collection (ATCC) 334* and described the anti-tumor mechanism of ferrichrome in colorectal cancer cells in vitro [17]. Ferrichrome activated the DNA damage inducible transcript 3 (DDIT3) signal and induced apoptosis in colorectal cancer cells. In addition, ferrichrome showed a less-marked growth inhibition effect in non-cancerous cells derived from the intestine, suggesting that ferrichrome may be an effective and safe drug for colorectal cancer treatment. Considering the drug positioning of ferrichrome, it is important to determine the appropriate phase of tumor progression, including precancerous phases, during which to use ferrichrome and to compare the anti-tumor effect and safety of ferrichrome with those of existing antitumor drugs.

In the present study, we used six colorectal cancer cell lines, an in vivo xenograft model and patient-derived organoids to confirm the anti-tumor effects of ferrichrome for sporadic colorectal neoplasms associated with the adenoma-carcinoma sequence pathway and used a dextran sulfate sodium (DSS)-colitis and azoxymethane (AOM)-DSS carcinogenesis mouse model to confirm the anti-tumor effects of ferrichrome on colitis-associated tumorigenesis. In addition, the anti-tumor effects of ferrichrome were compared with those of currently available baseline drugs, including 5-FU and cisplatin, and the combination effects of their drugs were analyzed in in vitro and in vivo xenograft models. In addition, the acute adverse events of ferrichrome were examined in order to support the potential clinical use of ferrichrome.

## Materials and methods

### Cell culture

Human cancer cell lines were grown in Roswell Park Memorial Institute (RPMI) 1640 (SW620 [RRID:CVCL_0547], SW480 [RRID:CVCL_0546]), high-glucose Dulbecco’s Modified Eagle’s Medium (DMEM) (C2BBE1 [Caco2/bbe] [RRID;CVCL_1096], HT-29 [RRID:CVCL_0320], SK-CO-1 [RRID:CVCL_0626], McCoy’s 5A Medium (HCT 116 [RRID:CVCL_0291)]supplemented with 10% (vol/vol) fetal bovine serum (FBS), 2 mM L-glutamine, 50 U/ml penicillin and 50 µg/ml streptomycin in a humidified atmosphere containing 5% CO_2_. All experiments were performed with mycoplasma-free cells. All human cell lines were authenticated using STR profiling within the last three years.

### Mice

BALB/c or BALB/c nude mice (6 weeks of age) were purchased from Sankyo Labo Service (Tokyo, Japan). The study received ethical approval for the use of an opt-out methodology from the Medical Ethics Committee of Asahikawa Medical University (Approval No. 16,069, 20,057).

### AOM-DSS carcinogenesis model

AOM (10 mg/kg) was intraperitoneally injected into the BALB/c mice. One week after the injection, the mice were treated with 1 or 1.5% DSS in the drinking water for 1 week. The body weight of mice was measured with time.

### Human intestinal epithelial cells

Biopsy tissue of colon adenoma and colon adenocarcinoma was obtained under colonoscopy. The experiments were undertaken with the understanding and written consent of each subject. The study methodologies conformed to the standards set by the Declaration of Helsinki. The study methodologies were approved by the Asahikawa Medical University Institutional Care and Use Committee (Approval No. 1668).

### Organoid culture

In a 15 mL conical tube, the tissue sample was washed with 10 mL of ice-cold phosphate-buffered saline (PBS). The tissue and remaining supernatant were then transferred to a 1.5 mL microcentrifuge tube. Using sterile scissors, the tissue was thoroughly minced into the smallest pieces possible. The tissue fragments were then transferred to a new 15 mL conical tube, and 10 mL of Gentle Cell Dissociation Reagent was added, followed by incubation on ice for 30 minutes. The sample was then centrifuged at 290 *g* for 5 minutes, after which the supernatant was aspirated, and 1 mL of ice-cold DMEM + 1% bovine serum albumin (BSA) was added. Using a 1 mL pipettor, the contents of the tube were passed through a 70-µm cell strainer into a new 15 mL conical tube. The sample was then centrifuged at 200 *g* for 5 minutes, after which all but 100 µL of the supernatant was aspirated. Matrigel® (100 µL) was added to the sample tube, and 50 µL of the Matrigel®-crypt suspension was placed on a 24-well plate. The plate was incubated at 37 °C for 10 minutes to allow the Matrigel®-crypt suspension to solidify, after which 750 µL of IntestiCult™ Organoid Growth Medium Y-27,632 (10 µM final concentration) was added for primary culture to each well.

### The assessment of the cell viability via 3-(4,5-dimethylthiazol-2-yl)-2,5-diphenyltetrazolium bromide (MTT) reduction

The viabilities of seeded organoids were normalized before apoptosis induction by incubation with resazurin. The organoids were cultured with 10 mg/ml resazurin (Sigma) for 6 h, and then the supernatant was transferred into a 96-well microplate and the fluorescence measured with a fluorescence detector at 530 nm excitation and 590 nm emission. Organoid growth was assessed using an MTT cell proliferation kit I (Roche Diagnostics GmbH, Mannheim, Germany). The organoids were seeded onto 96-well microplates at 100 to 500 per well for 96 h prior to stimulation. The organoids were subsequently incubated with 10 µL of MTT labeling reagent for 4 h, followed by the addition of 100 µL of solubilization solution into each well. The plates were left in the incubator overnight, and the optical density (OD) was measured at a 570 nm test wavelength and a 650 nm reference wavelength. Matrigel without organoids (7 µL) was used as the control and set as 0% viability. Untreated organoids were defined as 100% viability. If normalization with resazurin was done before treatment, the relative MTT reduction was divided by the fluorescence value of the respective wells [18].

### Ferrichrome

Ferrichrome was purchased from Sigma-Aldrich (St. Louis, MO, USA, Cat No; F8014).

### Sulforhodamine B (SRB) assay

The cells were seeded onto 96-well microplates at 1 × 10^4^ cells per well. Ferrichrome was diluted in DMEM and used to treat the cells. The cells were then fixed in 5% trichloroacetic acid (TCA) for 1 h at 4 °C and washed 4 times in distilled water. The microplates were then dehydrated at room temperature, stained in 100 µl/well of 0.057% (wt/vol) SRB powder/distilled water, washed 4 times in 0.1% acetic acid and re-dehydrated at room temperature. The stained cells were lysed in 10 mM Tris-buffer, and the OD was measured at 510 nm.

### Xenografts

The protocols of the animal experiments were approved by the Asahikawa Medical University Institutional Animal Care and Use Committee. SW620 or HCT116 cells (2 × 10^6^ cells) were injected into male BALB/c nude mice. Ferrichrome or 5-FU treatments were administered daily, starting the day after the injection of cancer cells. The tumor volumes were calculated from digital caliper raw data using the following formula: Volume = (major tumor diameter) × (minor tumor diameter)^2^/2.

### Real‐time polymerase chain reaction (RT-PCR)

Total RNA was extracted using an RNeasy mini kit (Qiagen, Tokyo, Japan) according to the manufacturer’s instructions. The mRNAs were reverse transcribed using a high-capacity cDNA reverse transcription kit (Applied Biosystems, Foster City, CA, USA). The cDNA was amplified using the specific primer for DDIT3 (purchased from Applied Biosystems), and signal detection was performed in triplicate using an Applied Biosystems 7300 Real-Time PCR System. The average mRNA expression was normalized to the 18S rRNA expression (Applied Biosystems).

### Terminal deoxynucleotidyl transferase dUTP nick‐end labeling (TUNEL) staining

The cells were plated onto a 96-well plate, fixed in 4% paraformaldehyde and washed extensively with PBS. The cells were stained using an In Situ Cell Death Detection Kit and TMR red (Roche Diagnostic, Indianapolis, IN, USA) according to the manufacturer’s instructions. After incubation with staining reagents, the samples were washed three times with PBS, and the TUNEL-positive cells were visualized by fluorescence microscopy (KEYENCE Corporation).

### Serum biochemistry

The serum of ferrichrome-treated mice was obtained from the inferior vena cava. Biochemical analyses were performed by Oriental Yeast Co., Ltd. (Japan).

### Statistical analyses

The assay data were analyzed using Student’s *t*-test and a one- or two-way ANOVA followed by Ryan’s correction. *P* values of < 0.05 were considered to indicate statistical significance.

### Data availability

The data that support the findings of this study are available from the corresponding author upon reasonable request.

## Results

### Ferrichrome inhibited the growth of six colorectal cancer cells in vitro and in vivo

**Fig. 1:**
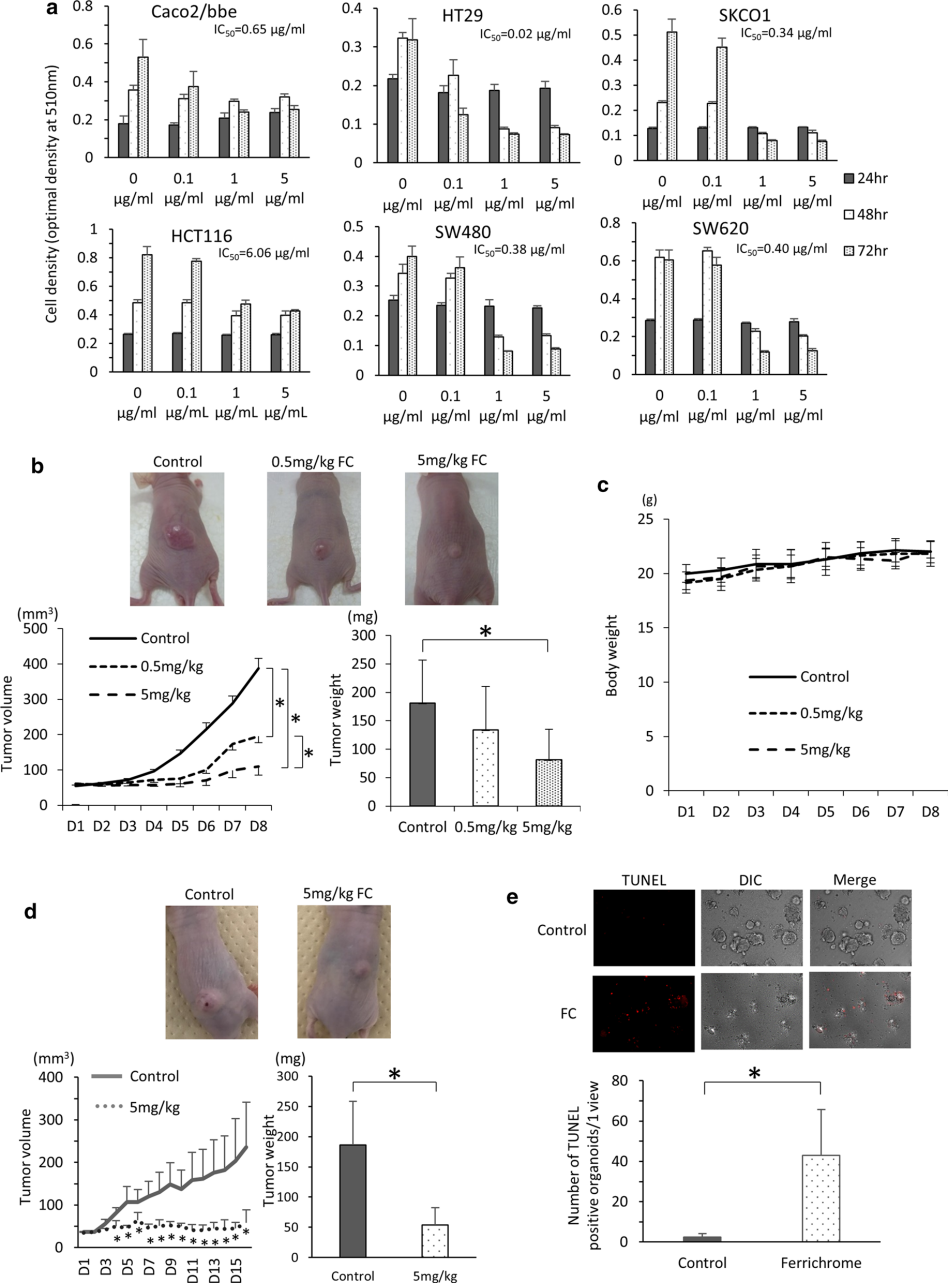
Ferrichrome reduced the progression of colorectal cancer cells derived from primary or metastatic lesions. An SRB assay revealed that the numbers of colorectal cancer cells (Caco2/bbe, HCT116, SW-480 and HT29 cells, SK-CO-1 and SW-620 cells) were significantly lower in the ferrichrome groups than in the control group (n = 6) (**a**). The tumor volume and weight **b** in mice transplanted with SW620 cells were significantly decreased by the intraperitoneal injection of 5 mg/kg of ferrichrome without a decrease in the body weight **c** (0 mg/kg; n = 7, 5 mg/kg; n = 6). The tumor volume and weight **d** of mice transplanted with HCT116 cells were significantly decreased by the intraperitoneal injection of 5 mg/kg of ferrichrome (0 mg/kg; n = 5, 5 mg/kg; n = 6). The proportion of TUNEL-positive organoids **e** was significantly increased in the ferrichrome group compared to the control group. *p < 0.05 by a two-way ANOVA followed by Ryan’s post hoc test (A, B, D). *p < 0.05 by Student’s *t*-test (**c**,** e**). The error bars show the standard deviation (S.D.)

To clarify the tumor-suppressive effect in colorectal cancer cells in vitro, ferrichrome was administered to Caco2/bbe, HT29, SK-CO-1, HCT116, SW480 and SW620 cells. The characteristics of each cell line are shown in Table 1. The growth of each cell line was suppressed by ferrichrome treatment (Fig. 1a).

**Table 1:** Characteristics of the colorectal cancer cells

Cell type	Age	Sex	Race	Derivation	Origin tissue
SW480	50	M	Caucasian	Primary lesion	Colon
SW620	51	M	Caucasian	Metastasis lesion	Lymph node
SKCO1	65	M	Caucasian	Metastasis lesion	Ascites
Caco2	72	M	Caucasian	Primary lesion	Colon
HCT116	adult	M		Primary lesion	Colon
HT29	44	F	Caucasian	Primary lesion	Colon

To assess the tumor-suppressive effect of ferrichrome in vivo, SW620 cells and HCT116 cells were transplanted to nude mice. The tumor volume and weight were significantly decreased by the intraperitoneal injection of 5 mg/kg ferrichrome to the SW620 tumor (Fig. 1b). However, no marked change in the body weight was observed on ferrichrome treatment (Fig. 1c). Likewise, a tumor-suppressive effect of the intraperitoneal injection of 5 mg/kg ferrichrome was observed for the HCT116 tumor (Fig. 1d), suggesting that ferrichrome exerts anti-tumor effects against colorectal cancer in vivo.

### Assessing the antitumor effect of ferrichrome in patient‐derived sporadic colorectal neoplasms

To clarify the anti-tumor effect of ferrichrome on cancer cells associated with the adenoma-carcinoma sequence pathway, 9 adenoma and 9 carcinoma organoids derived from patients were constructed from endoscopic biopsied specimens. The details of the patients who underwent endoscopic biopsies are described in Table 2. The MTT/resazurin assay revealed that the growth suppression rate of each ferrichrome-treated organoid was 68.4% (A1), 52.6% (A2), 67.4% (A3), 81.6% (A4), 56.1% (A5), 59.2% (A6), 55.9% (A7), 41.6% (A8), 45.7% (A9), 68.2% (C1), 52.3% (C2), 35.0% (C3), 85.3% (C4), - 7.7% (C5), 73.8% (C6), 73.0% (C7), 80.7% (C8) and 40.5% (C9) (Table 3) [The dose dependency was confirmed in A1 organoid (Additional file 1: Fig. S1)]. Ferrichrome significantly inhibited the growth in seven adenoma organoids (A1, A3, A4, A5, A6, A7, A8) and seven carcinoma organoids (C1, C2, C3, C4, C6, C7, C8) but not in the four other organoids (A2, A9, C5, C9).

We previously showed that ferrichrome induced colorectal cancer apoptosis through the activation of the DDIT3 signaling [17]. The change in the DDIT3 mRNA expression in the 18 ferrichrome-treated organoids was therefore assessed. Real-time PCR revealed the ratio of DDIT3 mRNA (ferrichrome treated organoids/ferrichrome un-treated organoids) to be 1.54 (A1), 1.62 (A2), 2.80 (A3), 1.89 (A4), 1.88 (A5), 1.95 (A6), 2.03 (A7), 1.39 (A8), 1.86 (A9), 2.28 (C1), 1.80 (C2), 4.21 (C3), 1.54 (C4), 1.04 (C5), 1.50 (C6), 3.07 (C7), 6.21 (C8), and 1.63 (C9) (Table 3). The expression of DDIT3 was significantly induced in 14 organoids treated with 1 mg/mL of ferrichrome (A1, A2, A3, A4, A5, A7, A8, C1, C2, C3, C4, C7, C8, C9) (*p* < 0.05) and tended to be induced in the remaining 1 organoid (A6) (p = 0.0626), in which ferrichrome significantly inhibited the tumor growth in the MTT assay. In contrast, DDIT3 was not induced in the three organoids (A9, C5, C6) in which ferrichrome exhibited no inhibitory effect in the MTT assay. This suggests that the ferrichrome-induced tumor inhibition was mediated by the upregulation of DDIT3. To confirm whether or not ferrichrome induced apoptosis in patient-derived sporadic colorectal cancer, a TUNEL assay was performed in ferrichrome-treated organoids. The proportion of TUNEL-positive organoids was significantly higher in the ferrichrome group than in the control group (Fig. 1e). These results suggest that ferrichrome induces apoptosis and thus suppresses the growth of sporadic colorectal neoplastic cells of organoids.

**Table 2:** Characteristics of the patient-derived adenoma and carcinoma organoids

No	Age	Sex	Pathology	Tumor site	Tumor size(mm)	Macroscopic type	IC50(mg/ml)
A1	71	M	Low grade tubular adenoma	Sigmoid colon	12	0-Is	0.47
A2	74	F	Mixed low and high tubular adenoma	Transverse colon	6	0-Is	0.87
A3	87	M	Mixed low and high tubular adenoma	Transverse colon	12	0-Isp	0.55
A4	38	M	Sessile serrated adenoma/polyp	Cecum	15	0-IIa	0.32
A5	63	M	High tubular adenoma	Rectum	7	0-Isp	0.86
A6	71	M	Low grade tubular adenoma	Transverse colon	11	0-Isp	0.64
A7	47	M	Sessile serrated adenoma/polyp	Decending colon	10	0-Is	0.4
A8	66	M	Low grade tubular adenoma	Ascending colon	6	0-Is	1.56
A9	66	M	Sessile serrated adenoma/polyp	Transverse colon	5	0-Is	1.97
C1	76	F	Moderately	Rectum	Stage I (T1b,N0,MO)	2	0.43
C2	73	F	Well > moderately	Transverse colon	Stage IVa (T4a,N2a,M1a)	2	0.71
C3	57	M	Mucinous > > moderately	Rectum	Stage IIIb (T3,N1b,M0)	2	2.2
C4	87	M	Well	Ascending colon	Stage 0(Tis,N0,MO)	0-Isp	0.71
C5	51	M	Well	Rectum	Stage I (T1b,N0,MO)	2	N.D
C6	71	M	Well > moderately	Ascending colon	Stage 0 (Tis,N0,MO)	0-Ip	0.6
C7	71	M	Moderately > > poorly	Rectum	Stage IIA (T3,N0,M0)	2	0.48
C8	82	M	Well > moderately	Ascending colon	Stage II (T2,N0,M0)	2	0.22
C9	73	F	Moderately > well	Rectum	Stage IVa (T3,N0,M1a)	2	3

**Table 3:** The growth suppression rate and fold change of ddit3 mRNA in ferrichrome-administered organoids

	Suppression rate(%)	*p* value (growth suppression rate)	DDIT3 FC/control	*p* value(fold change of DDIT3)
A1	68.4 ± 6.4	0.00621	1.54 ± 0.34	0.0274
A2	52.6 ± 16.4	0.15	1.62 ± 0.19	0.0000873
A3	67.4 ± 14.1	0.000512	2.8 ± 1.4	0.0222
A4	81.6 ± 2.9	0.000813	1.89 ± 0.33	0.015
A5	56.1 ± 13.2	0.00158	1.88 ± 0.29	0.00137
A6	59.2 ± 6.1	0.00396	1.95 ± 0.61	0.0626
A7	55.9 ± 1.9	0.0381	2.03 ± 0.48	0.021
A8	41.6 ± 15	0.00176	1.39 ± 0.11	0.0169
A9	45.7 ± 9.8	0.0623	1.86 ± 0.54	0.124
C1	68.2 ± 5	0.0122	2.28 ± 0.72	0.0376
C2	52.3 ± 12.3	0.0165	1.8 ± 0.62	0.0274
C3	35 ± 0.5	0.00796	4.21 ± 0.86	0.00622
C4	85.3 ± 2.2	0.0000361	1.54 ± 0.25	0.022
C5	− 7.7 ± 15	0.584	1.04 ± 0.16	0.719
C6	73.8 ± 58	0.0114	1.50 ± 0. 0.97	0.426
C7	73 ± 12.3	0.0142	3.07 ± 0.38	0.00000437
C8	80.7 ± 8.5	0.000000443	6.21 ± 1.90	0.00913
C9	40.5 ± 23	0.25	1.63 ± 0.08	0.00395

### Ferrichrome exerted an anti‐tumor effect in a colitis‐associated cancer model in vivo

**Fig. 2:**
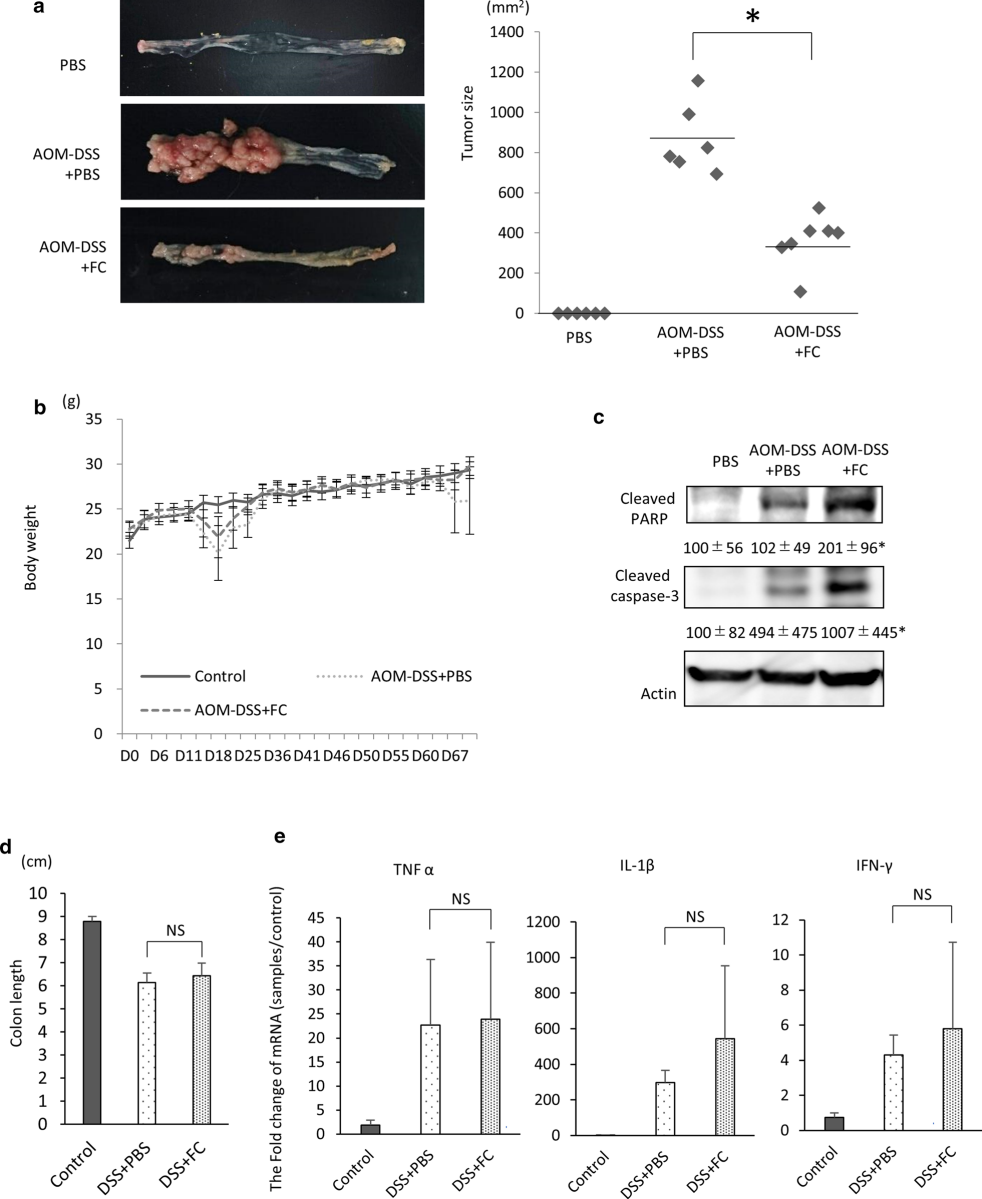
Ferrichrome exerted anti-tumor effects but not the anti-inflammatory effects in an in vivo animal model. Ferrichrome (5 mg/kg) was administered via intraperitoneal injection to AOM-DSS carcinogenesis model mice for 69 days. The tumor size in the ferrichrome-treated model mice was significantly reduced **a** without any marked change in the body weight (**b**). A Western blot analysis showed that the accumulation of cleaved caspase-3 and PARP was significantly higher in the ferrichrome-treated group than in the control group (**c**). However, 5 mg/kg of ferrichrome administered trans-anally did not improve the colon length d or the expression of inflammatory cytokines (TNFα, IL1β and IFNγ) e in a DSS-colitis model. *p < 0.05 by a one-way ANOVA followed by Ryan’s post hoc test (**a**). *p < 0.05 by Student’s *t*-test (**b**, **d**, **e**). The error bars show the standard deviation (S.D.)

To investigate the anti-tumor effect of ferrichrome on colorectal cancer associated with the colitis-associated cancer pathway, an AOM-DSS carcinogenesis mouse model was constructed. Ferrichrome (5 mg/kg) was intraperitoneally administered every other day. The tumor size was significantly reduced by the administration of ferrichrome in the AOM-DSS carcinogenesis mouse model (Fig. 2a). No significant change in the body weight was noted in the AOM-DSS + ferrichrome or AOM-DSS + PBS group (Fig. 2b). A Western blotting analysis showed the accumulation of cleaved caspase-3 and poly(ADP-ribose)polymerase (PARP) in the AOM-DSS + ferrichrome group compared to the AOM-DSS + PBS group, suggesting that tumor cell apoptosis had been induced by ferrichrome treatment (Fig. 2c).

To clarify the ferrichrome effect on the precancerous condition of colitis-associated cancer, a DSS-colitis mouse model was constructed. Ferrichrome treatment did not change the colon length or expression of proinflammatory cytokines, including TNF alpha, IFN-gamma and IL-1beta (Fig. 2d, e). These data indicated that ferrichrome exerts an anti-tumor effect on colitis-associated cancer but not on the precancerous, non-neoplastic phase of the pathway.

### The anti‐tumor effect of ferrichrome was stronger than that of 5-FU and cisplatin

**Fig. 3:**
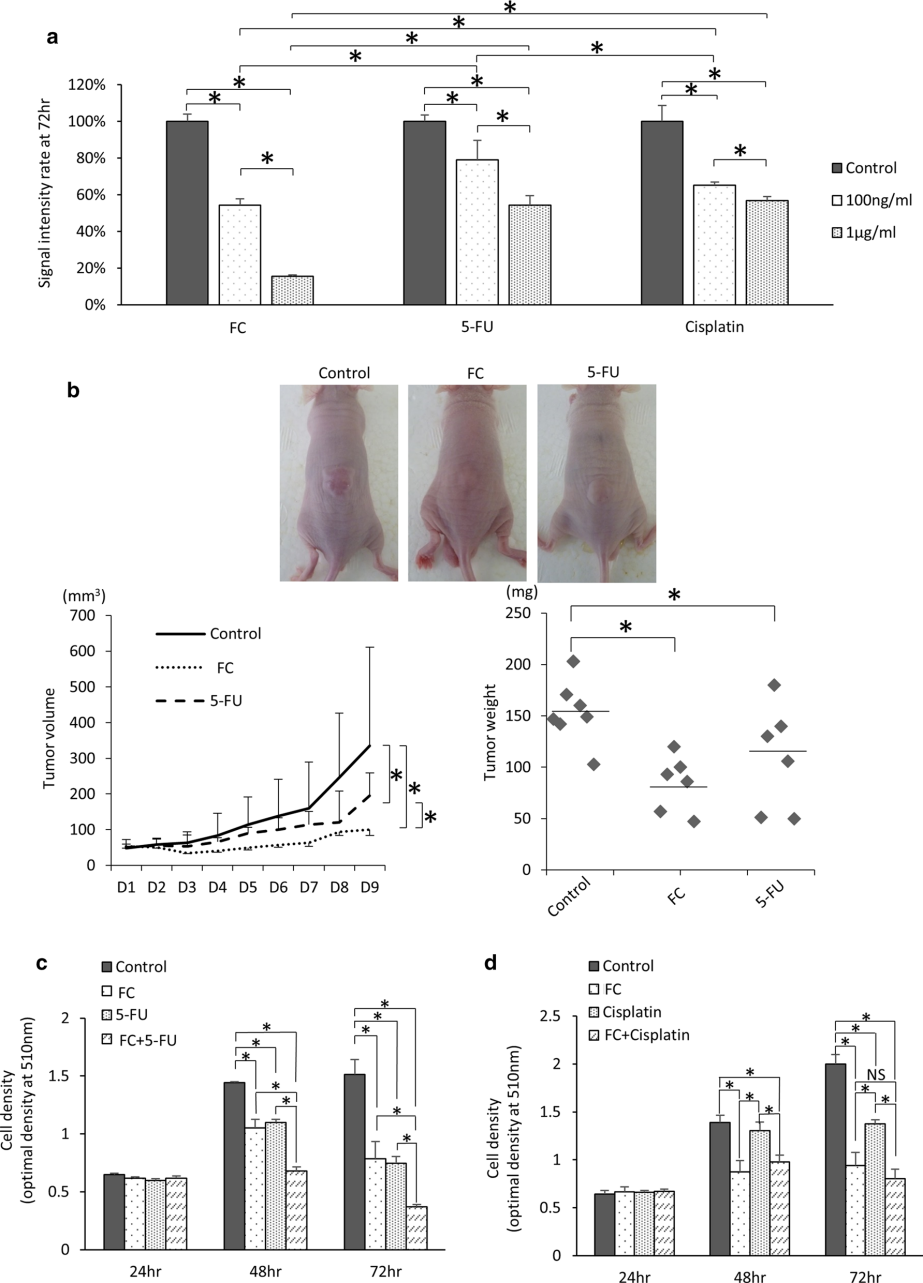
The anti-tumor effect of ferrichrome was greater than that of 5-FU or cisplatin. An SRB assay for SW620 revealed that the tumor-suppressive effect of ferrichrome was superior to that of 5-FU and cisplatin (**a**). Ferrichrome (0.5 mg/kg) and 5-FU (0.5 mg/kg) significantly suppressed the tumor growth in comparison to the control, and the tumor volume of the ferrichrome treated group also significantly decreased in comparison to the 5-FU treated group (**b**). An SRB assay showed that the anti-tumor effect of 0.2 µg/mL of ferrichrome was equal or greater than that of 0.2 µg/mL of 5-FU or cisplatin, and the combination of ferrichrome and 5-FU greatly reduced the density of SW620 cells compared to treatment with 5-FU alone (**c**, **d**). *p < 0.05 by a two-way ANOVA followed by Ryan’s post hoc test. The error bars show the standard deviation (S.D.)

To compare the anti-tumor effect of ferrichrome and currently available anti-tumor agents, SW620 cells were treated with these agents. An SRB assay revealed that the tumor-suppressive effect of ferrichrome was superior to that of 5-FU and cisplatin (Fig. 3a). To assess the antitumor effects of ferrichrome and 5-FU *in vivo*, a mouse xenograft model of SW620 cells was constructed, and experimental agents were directly injected into the tumor. The tumor volume and weight were significantly suppressed by the administration of either 0.5 mg / kg of ferrichrome or 5-FU in comparison to the control group, and the tumor suppressive effect of ferrichrome was superior to that of 5-FU (Fig. 3b). These data indicated that ferrichrome exerted a strong tumor-suppressive effect, and its effect was superior to that of currently available anti-tumor agents, including 5-FU and cisplatin, both in vitro and in vivo*.*

### The effect of ferrichrome in combination with 5-FU is stronger that of 5-FU alone in vivo

**Fig. 4:**
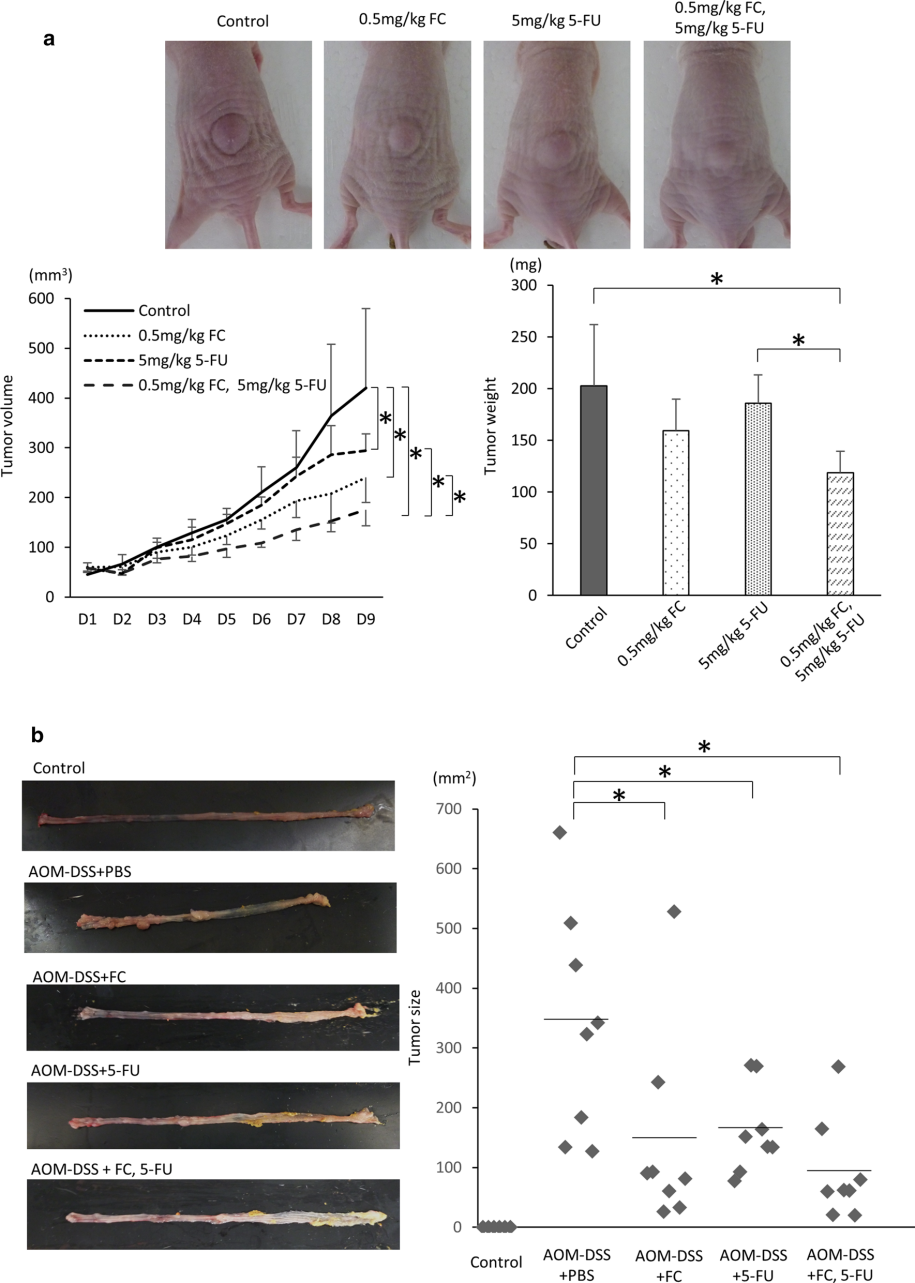
The anti-tumor effect of the combination of ferrichrome and 5-FUwas greater than that of 5-FU alone.The tumor-suppressive effects of the combination of 0.5 mg/kg ferrichrome and 5 mg/kg 5-FU, including changes in the tumor volume and tumor weight, were significantly superior to those of monotherapy with these agents in a SW620 transplanted xenograft mouse model (**a**). Likewise, ferrichrome (5 mg/kg) and 5-FU (5 mg/kg) were administered to the AOM-DSS carcinogenesis model mice by intraperitoneal injection. The average tumor size of the combination treatment group was smaller in comparison to the single treatment group (**b**). *p < 0.05 by a one-way ANOVA followed by Ryan’s post hoc test. The error bars show the standard deviation (S.D.)

The prognosis of colorectal cancer patients with an insufficient response to 5-FU or cisplatin treatment is poor, so enhancing the effect of 5-FU is clinically important for improving the outcome of such patients. To assess the combination effect of ferrichrome and currently available anti-tumor agents, the mixture of ferrichrome and cisplatin or 5-FU was administered to SW620 cells. The SRB assay revealed that the anti-tumor effect of low-dose (0.2 µg/mL) ferrichrome was equal to or stronger than that of low-dose (0.2 µg/mL) 5-FU or cisplatin. The combination of ferrichrome and 5-FU highly suppressed the tumor growth compared to 5-FU alone, but the combination of ferrichrome and cisplatin showed no additional anti-tumor effect (Fig. 3c, d). Ferrichrome (0.5 mg/kg) and 5-FU (5 mg/kg) were then intraperitoneally injected into SW620 transplanted nude mice. The concentration of 5-FU was determined based on the FOLFOX regimen generally used in colorectal cancer patients. The tumor volume and weight were reduced by treatment with ferrichrome or 5-FU. The tumor-suppressive effect was significantly augmented by the combination of ferrichrome and 5-FU compared to single-agent administration of either one (Fig. 4a). Likewise, ferrichrome (5 mg/kg) and 5-FU (5 mg/kg) were intraperitoneally administered to the AOM-DSS carcinogenesis model mice. The average tumor size of the combination treatment group was smaller in comparison to the single treatment group (Control; 399.8 ± 190.2, Ferrichrome; 144.5 ± 168.9, 5-FU; 162.0 ± 72.7, ferrichrome and 5-FU; 92.2 ± 84.4), suggesting an additional effect of ferrichrome on colorectal cancer cells when the effect of 5-FU is insufficient (Fig. 4b).

### Ferrichrome is an anti‐tumor molecule with few adverse effects

**Fig. 5:**
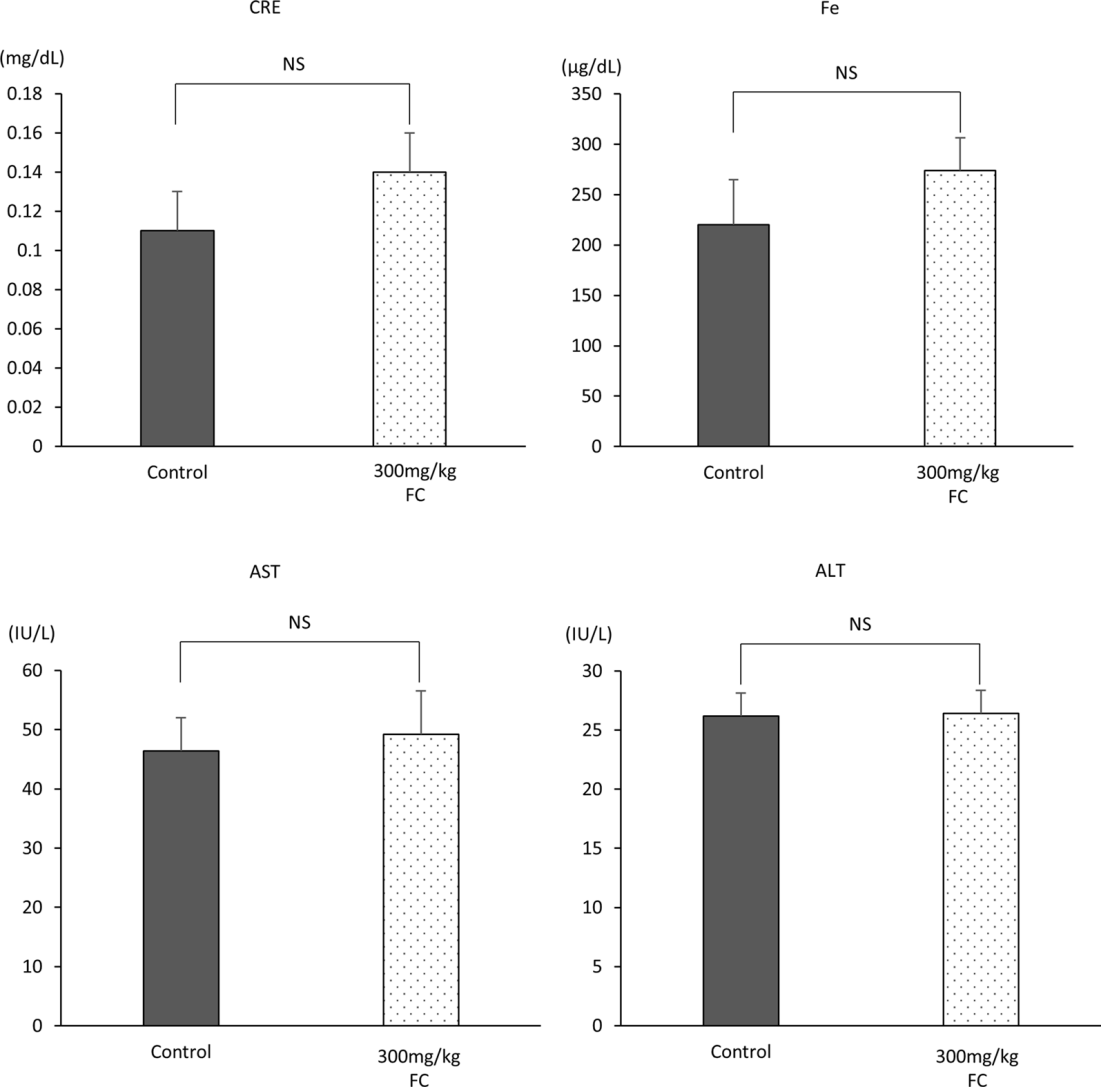
The in vivo acute toxicity level of intravenously administered ferrichrome. The serum was collected from the inferior vena cava of 300 mg/kg ferrichrome-treated mice, and biochemical tests were performed. There were no significant changes in the test values of AST, ALT, creatinine or iron. *p < 0.05 by Student’s *t*-test. The error bars show the standard deviation (S.D.)

To assess the acute toxicity of excessive amounts of ferrichrome administration, 300 mg/kg ferrichrome, which is 60-fold the anti-tumor amount used in the AOM-DSS and tumor-transplanted models, was administered to the mice via tail vein injection. There were no dead mice on day 1 of observation (data not shown). Serum was then collected from the inferior vena cava of 300 mg/kg ferrichrome-treated mice, and biochemical tests were performed. There were no significant changes in the test values of AST, ALT or creatinine. Furthermore, we assessed the serum iron contents because ferrichrome is an Fe^3+^ chelator derived from bacteria [19]. However, the iron content was not markedly changed by ferrichrome treatment (Fig. 5). Therefore, the acute toxic level of ferrichrome is thought to be over 300 mg/kg via intravenous administration in vivo.

## Discussion

The present study investigated the anti-tumor effect of ferrichrome, which was detected in the conditioned media of the probiotic *L. casei* as a mediator of the bacterial anti-tumor function, on colorectal cancer model using the patient-derived organoids and colitis-associated carcinogenesis model. Of note, ferrichrome exerted a greater tumor-inhibitory effect than currently available antitumor drugs, including 5-FU and cisplatin, both in vitro and in a xenograft model, and there were no adverse effects on the biochemical test values of AST, ALT, CRE and Fe in single dose toxicity test, indicating that ferrichrome is a feasible treatment agent for colorectal tumors with a strong anti-tumor effect and broad safety range.

First, to assess the anti-tumor effect of ferrichrome on sporadic colorectal cancer, an SRB assay of six representative colorectal cancer cell lines (Caco2/bbe, HT29, SKCO1, HCT116, SW480 and SW620 cells) was performed. The assay showed that ferrichrome exerted significant anti-tumor effects for the six cell lines. An in vivo xenograft mouse model confirmed the anti-tumor effect of ferrichrome on SW620 cells and HCT116. Subsequently, patient-derived adenoma and cancer organoids were obtained to confirm the anti-tumor effect of ferrichrome on sporadic colorectal neoplasms. An MTT with resazurin assay revealed that ferrichrome inhibited the tumor growth in seven of the nine adenoma organoids and seven of the nine carcinoma organoids. These findings show that ferrichrome exerts an anti-tumor effect on sporadic colorectal neoplasms, including precancerous adenoma as well as cancer cells that may be associated with adenoma-carcinoma sequence. Real-time PCR of DDIT3 revealed that ferrichrome exerted an anti-tumor effect in 12 of 14 patient-derived organoids in a DDIT3 induction-dependent manner. A previous investigation showed that DDIT3 is a key transcriptional factor that induces the apoptotic pathway [21, 22] by binding the promoter of death receptors (DRs), including DR4 and DR5, to induce apoptosis through the BAX-BAK-mediated mitochondrial pathways [23], thus illustrating that the anti-tumor effect of ferrichrome is mediated by the induction of DDTI3 in cancer cells. However, ferrichrome did not exert a significant tumor-suppressive effect in the A2 or C9 organoids (A2: 52.6%, p = 0.15, C9: 40.5%, p = 0.25) despite significantly inducing the DDIT3 expression (A2: 1.62, C9: 1.63). The promoter region of DR4 is reportedly frequently methylated, leading to TRAIL-induced apoptosis in lung squamous carcinoma cells [24]. It is speculated that the promoter regions of DDIT3 may be modulated through genetic or epigenetic modification in A2 and C9 organoids, resulting in a low tumor-suppressive effect despite the significant induction of DDIT3 when treated with ferrichrome.

Second, to assess the anti-tumor effect of ferrichrome on cancer cells associated with the colitis-associated pathway, an AOM-DSS carcinogenesis model was constructed. The AOM-DSS model showed that ferrichrome greatly reduced the tumor area, and Western blotting showed that the cleavage of caspase 3 and PARP was significantly induced in the ferrichrome treated AOM-DSS model, illustrating the anti-tumor effect of ferrichrome through the induction of apoptosis on colorectal cancer cells associated with the colitis-associated pathway. In addition, ferrichrome did not influence the expression of proinflammatory cytokines, including TNF-alpha, IFN-gamma and IL-1beta, and the histological severity in a DSS-colitis model, thus indicating that ferrichrome inhibits cancer cell growth, but not the advent of a precancerous condition, such as inflammation, related to the colitis-associated pathway.

The tumor-suppressive effect of the combination of ferrichrome and 5-FU was superior to that of single treatment of each drug in vitro and in vivo xenograft model of SW620 cells and AOM-DSS carcinogenesis model mice. Previous our investigation revealed that ferrichrome induces the apoptosis through up-regulating the DDIT3 signal cascade and thereby activating the caspase-3 pathway, inducing DNA fragmentation. In contrast, 5-FU exerts its tumor-suppressing effect through two pathways, including the blockage of thymidylate synthase and the inhibition of RNA processing due to the incorporation of fluorouracil uracil triphosphate into the RNA sequence instead of uridine triphosphate. This suggests that ferrichrome-induced DDIT3 targets tumor cell DNA, while 5-FU targets DNA as well as other targets, such as RNA processing [26]. The differences in the antitumor mechanisms between ferrichrome and 5-FU resulted in an additive effect on colorectal cancer therapy, so ferrichrome can be used as an anti-tumor drug in cancer patients for whom 5-FU and cisplatin show insufficient efficacy or strong side effects. Conversely, the combination of ferrichrome and cisplatin did not exert any additive effects compared with single treatment of each drug. Cisplatin is known to target DNA through bridging between guanine and adenine, inhibiting DNA duplication [27]; it also induces DDIT3-mediated apoptosis [28]. These findings suggest that either cisplatin or ferrichrome targets tumor cell DNA, leading to fewer additional effects of the combination of these two drugs.

## Conclusions

In conclusion, we showed that ferrichrome exerted anti-tumor effects on colorectal cancer that may be associated with the adenoma-carcinoma sequence and colitis-associated pathways. The anti-tumor effect of ferrichrome was mediated by the upregulation of DDIT3, and superior to that of 5-FU or cisplatin. The tumor-suppressive effect of the combination of ferrichrome and 5-FU was superior to that of single treatment of each drug in vitro and in vivo. The single-dose toxicity studies showed a sufficient safety of ferrichrome. These findings highlight ferrichrome derived from *L. casei* as an attractive candidate anti-cancer drug for colorectal cancer.

## Supplementary Information

**Additional file 1.** MTT assay showed the anti-tumor effect of ferrichrome in A1 organoid.

## Data Availability

All data generated or analyzed during this study are included in this published article.

## Abbreviations

DNA: Deoxyribonucleic acid
SRB: Sulforhodamine B
MTT: 3-(4,5-dimethylthiazol-2-yl)-2,5-diphenyltetrazolium bromide
AOM-DSS: Azoxymethane-dextran sulfate sodium
5-FU: 5-fluorouracil
APC: Adenomatous polyposis coli
DCC: Deleted in Colorectal Cancer
ATCC: American Type Culture Collection
JNK: The c-jun N-terminal kinase
DDIT3: DNA damage inducible transcript 3
RPMI: Roswell Park Memorial Institute
DMEM: Dulbecco’s Modified Eagle’s Medium
FBS: Fetal bovine serum
CO_2_: Carbon dioxide
BSA: Bovine serum albumin
Y27632: The Rho-associated protein kinase inhibitor
OD: Optical density
TCA: Trichloroacetic acid
PCR: Polymerase chain reaction
RNA: Ribonucleic acid
PARP: Polyadenosine diphosphate ribose polymerase
TNF: Tumor necrosis factor
IFN: Interferon
IL: Interleukin
AST: Aspartic aminotransferase
ALT: Alanine aminotransferase
Fe: Ferrum
DR: Death receptor
BAX: Bcl-2-associated X protein
BAK: Bcl-2 homologous antagonist/killer
TRAIL: TNF-related apoptosis-inducing ligand
IC_50_: The half-inhibitory concentration
FC: Ferrichrome
CRE: Creatinine
NS: Not significant

## References

[1] Sobrero AF, Maurel J, Fehrenbacher L, Scheithauer W, Abubakr YA, Lutz MP, Vega-Villegas ME, Eng C, Steinhauer EU, Prausova J, Lenz HJ, Borg C, Middleton G, Kröning H, Luppi G, Kisker O, Zubel A, Langer C, Kopit J, Burris HA (2008). EPIC: phase III trial of cetuximab plus irinotecan after fluoropyrimidine and oxaliplatin failure in patients with metastatic colorectal cancer. J Clin Oncol.

[2] Jonker DJ, O’Callaghan CJ, Karapetis CS, Zalcberg JR, Tu D, Au HJ, Berry SR, Krahn M, Price T, Simes RJ, Tebbutt NC, van Hazel G, Wierzbicki R, Langer C, Moore MJ (2007). Cetuximab for the treatment of colorectal cancer. N Engl J Med.

[3] Peták I, Schwab R, Orfi L, Kopper L, Kéri G (2010). Integrating molecular diagnostics into anticancer drug discovery. Nat Rev Drug Discov.

[4] Lund CM, Vistisen KK, Dehlendorff C, Rønholt F, Johansen JS, Nielsen DL (2017). The effect of geriatric intervention in frail elderly patients receiving chemotherapy for colorectal cancer: a randomized trial (GERICO). BMC Cancer.

[5] Kim JH (2015). Chemotherapy for colorectal cancer in the elderly. World J Gastroenterol.

[6] Hoeben KW, van Steenbergen LN, van de Wouw AJ, Rutten HJ, van Spronsen DJ, Janssen-Heijnen ML (2013). Treatment and complications in elderly stage III colon cancer patients in the Netherlands. Ann Oncol.

[7] Brenner H, Kloor M, Pox CP (2014). Colorectal cancer. Lancet.

[8] Lonardi S, Sobrero A, Rosati G, Di Bartolomeo M, Ronzoni M, Aprile G, Massida B, Scartozzi M, Banzi M, Zampino MG, Pasini F, Marchetti P, Cantore M, Zaniboni A, Rimassa L, Ciuffreda L, Ferrari D, Barni S, Zagonel V, Maiello E, Rulli E, Labianca R (2016). TOSCA (Three or Six Colon Adjuvant) Investigators. Phase III trial comparing 3–6 months of adjuvant FOLFOX4/XELOX in stage II-III colon cancer: safety and compliance in the TOSCA trial. Ann Oncol.

[9] Leslie A, Carey FA, Pratt NR, Steele RJ (2002). The colorectal adenoma-carcinoma sequence. Br J Surg.

[10] Coussens LM, Werb Z (2002). Inflammation and cancer. Nature.

[11] Sedivy R, Wolf B, Kalipciyan M, Steger GG, Karner-Hanusch J, Mader RM (2000). Genetic analysis of multiple synchronous lesions of the colon adenoma-carcinoma sequence. Br J Cancer.

[12] Leedham SJ, Graham TA, Oukrif D, McDonald SA, Rodriguez-Justo M, Harrison RF, Shepherd NA, Novelli MR, Jankowski JA, Wright NA (2009). Clonality, founder mutations, and field cancerization in human ulcerative colitis-associated neoplasia. Gastroenterology..

[13] Kang M, Martin A (2017). Microbiome and colorectal cancer: Unraveling host-microbiota interactions in colitis-associated colorectal cancer development. Semin Immunol.

[14] Sears CL, Garrett WS (2014). Microbes, microbiota, and colon cancer. Cell Host Microbe.

[15] Marteau PR, de Vrese M, Cellier CJ, Schrezenmeir J (2001). Protection from gastrointestinal diseases with the use of probiotics. Am J Clin Nutr.

[16] van Baarlen P, Wells JM, Kleerebezem M (2013). Regulation of intestinal homeostasis and immunity with probiotic lactobacilli. Trends Immunol.

[17] Konishi H, Fujiya M, Tanaka H, Ueno N, Moriichi K, Sasajima J, Ikuta K, Akutsu H, Tanabe H, Kohgo Y (2016). Probiotic-derived ferrichrome inhibits colon cancer progression via JNK-mediated apoptosis. Nat Commun.

[18] Grabinger T, Luks L, Kostadinova F, Zimberlin C, Medema JP, Leist M, Brunner T (2014). Ex vivo culture of intestinal crypt organoids as a model system for assessing cell death induction in intestinal epithelial cells and enteropathy. Cell Death Dis..

[19] Chu BC, Garcia-Herrero A, Johanson TH, Krewulak KD, Lau CK, Peacock RS, Slavinskaya Z, Vogel HJ (2010). Siderophore uptake in bacteria and the battle for iron with the host; a bird’s eye view. Biometals..

[20] Nambiar PR, Giardina C, Guda K, Aizu W, Raja R, Rosenberg DW (2002). Role of the alternating reading frame (P19)-p53 pathway in an in vivo murine colon tumor model. Cancer Res.

[21] Papadakis ES, Finegan KG, Wang X, Robinson AC, Guo C, Kayahara M, Tournier C (2006). The regulation of Bax by c-Jun N-terminal protein kinase (JNK) is a prerequisite to the mitochondrial-induced apoptotic pathway. FEBS Lett.

[22] Puthalakath H, O’Reilly LA, Gunn P, Lee L, Kelly PN, Huntington ND, Hughes PD, Michalak EM, McKimm-Breschkin J, Motoyama N, Gotoh T, Akira S, Bouillet P, Strasser A (2007). ER stress triggers apoptosis by activating BH3-only protein Bim. Cell.

[23] Hu H, Tian M, Ding C, Yu S (2019). The C/EBP Homologous Protein (CHOP) Transcription Factor Functions in Endoplasmic Reticulum Stress-Induced Apoptosis and Microbial Infection. Front Immunol.

[24] Wang W, Qi X, Wu M (2015). Effect of DR4 promoter methylation on the TRAIL-induced apoptosis in lung squamous carcinoma cell. Oncol Rep.

[25] Ijiri M, Fujiya M, Konishi H, Tanaka H, Ueno N, Kashima S, Moriichi K, Sasajima J, Ikuta K, Okumura T (2017). Ferrichrome identified from Lactobacillus casei ATCC334 induces apoptosis through its iron-binding site in gastric cancer cells. Tumour Biol.

[26] Longley DB, Harkin DP, Johnston PG (2003). 5-fluorouracil: mechanisms of action and clinical strategies. Nat Rev Cancer.

[27] Goodsell DS (2006). The molecular perspective: Cisplatin. Stem Cells.

[28] Huang Y, Chuang AY, Romano RA, Liégeois NJ, Sinha S, Trink B, Ratovitski E, Sidransky D (2010). Phospho-DeltaNp63alpha/NF-Y protein complex transcriptionally regulates DDIT3 expression in squamous cell carcinoma cells upon cisplatin exposure. Cell Cycle.

