# Supplementary figures and images for: Bacteria‐derived ferrichrome inhibits tumor progression in sporadic colorectal neoplasms and colitis‐associated cancer
Source: Cancer Cell Int. 2021 Jan 6;21:21. doi: 10.1186/s12935-020-01723-9 (PMC7789586; doi:10.1186/s12935-020-01723-9)

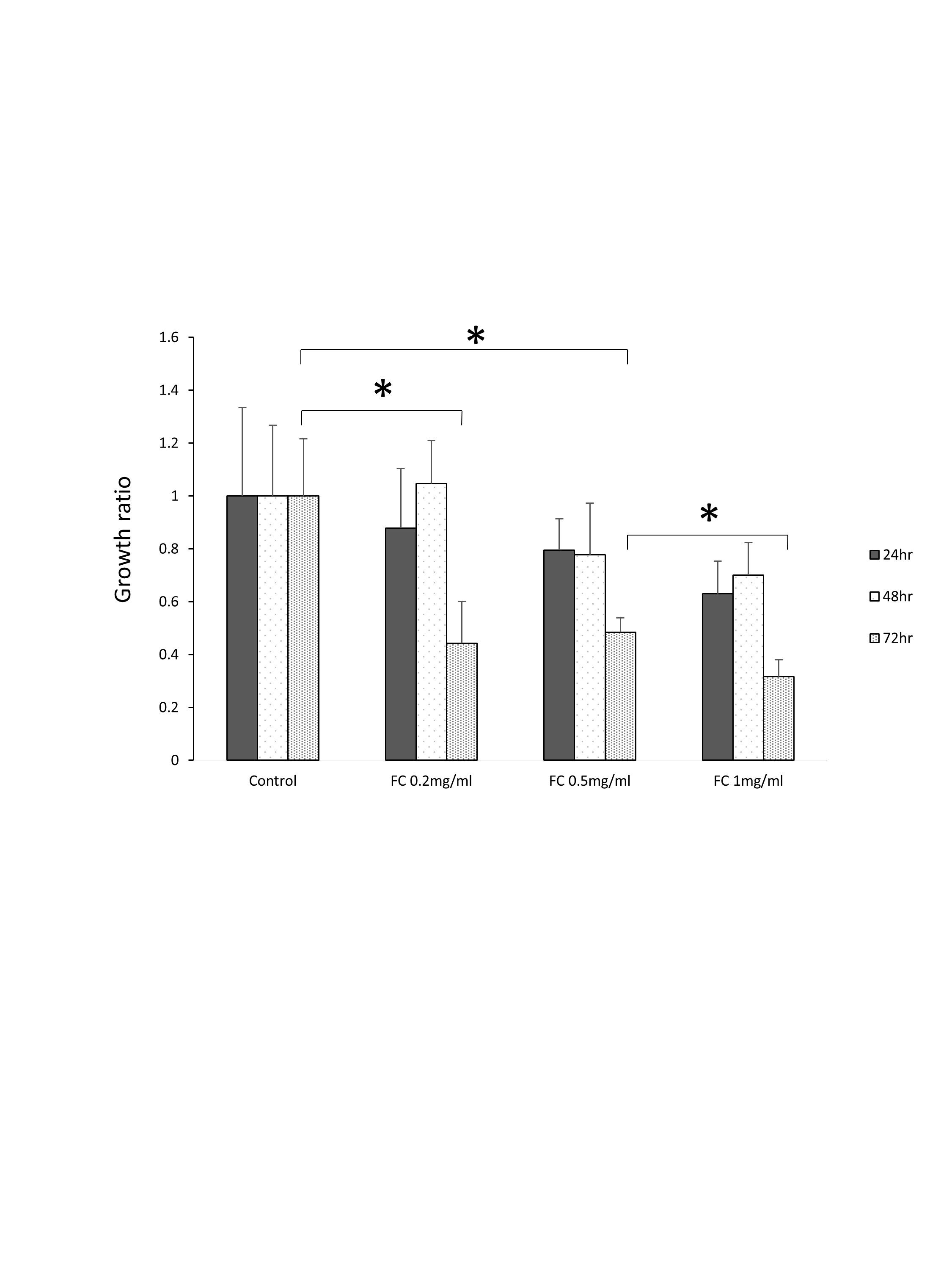

Supplement: Supplementary file 1 — Additional file 1. MTT assay showed the anti-tumor effect of ferrichrome in A1 organoid. [file 12935_2020_1723_MOESM1_ESM.tif]
